# Eribulin rapidly inhibits TGF-β-induced Snail expression and can induce Slug expression in a Smad4-dependent manner

**DOI:** 10.1038/s41416-019-0556-9

**Published:** 2019-09-04

**Authors:** Roma Kaul, April L. Risinger, Susan L. Mooberry

**Affiliations:** 1 Department of Pharmacology, University of Texas Health Science Center at San Antonio, San Antonio, TX USA; 2 Mays Cancer Center, University of Texas Health Science Center at San Antonio, San Antonio, TX USA

**Keywords:** Biomarkers, Molecular medicine

## Abstract

**Background:**

Evidence shows that the anticancer effects of microtubule targeting agents are not due solely to their antimitotic activities but also their ability to impair microtubule-dependent oncogenic signalling.

**Methods:**

The effects of microtubule targeting agents on regulators of TGF-β-induced epithelial-to-mesenchymal transition (EMT) were evaluated in breast cancer cell lines using high content imaging, gene and protein expression, siRNA-mediated knockdown and chromatin immunoprecipitation.

**Results:**

Microtubule targeting agents rapidly and differentially alter the expression of Snail and Slug, key EMT-promoting transcription factors in breast cancer. Eribulin, vinorelbine and in some cases, ixabepalone, but not paclitaxel, inhibited TGF-β-mediated Snail expression by impairing the microtubule-dependent nuclear localisation of Smad2/3. In contrast, eribulin and vinorelbine promoted a TGF-β-independent increase in Slug in cells with low Smad4. Mechanistically, microtubule depolymerisation induces c-Jun, which consequently increases Slug expression in cells with low Smad4.

**Conclusion:**

These results identify a mechanism by which eribulin-mediated microtubule disruption could reverse EMT in preclinical models and in patients. Furthermore, high Smad4 levels could serve as a biomarker of this response. This study highlights that microtubule targeting drugs can exert distinct effects on the expression of EMT-regulating transcription factors and that identifying differences among these drugs could lead to their more rational use.

## Background

Microtubule targeting agents are highly effective anticancer drugs that remain a mainstay in the treatment of a wide variety of cancers.^1^ Microtubule targeting agents are classified as either microtubule stabilizers, which increase the density of cellular microtubules, or microtubule destabilizers, which cause net microtubule loss.^2^ Microtubule stabilizing drugs, including the taxanes and ixabepilone, as well as the microtubule destabilizing vinca alkaloids and eribulin have clinical efficacy in breast cancer.^1^ All microtubule targeting agents disrupt microtubule dynamics and structure, thus inhibiting microtubule-dependent interphase and mitotic events. Growing evidence demonstrates that in addition to their antimitotic effects, microtubule targeting agents impair intracellular transport and trafficking in interphase cells resulting in disruption of oncogenic signalling.^3–8^ Key oncogenic proteins that traffic along microtubules include p53, Myc, Smad2/3, Rb, and Src.^4^ Microtubule targeting agents inhibit androgen receptor trafficking to the nucleus,^5,6^ nuclear transport of DNA repair proteins following DNA damage^7^ and prevent E-cadherin internalisation in a Src-dependent manner.^8^ The ability of microtubule targeting agents to interrupt a wide range of oncogenic signalling pathways likely contributes to their anticancer effects.

Given the differences among microtubule targeting agents with regard to their distinct tubulin/microtubule-binding sites and allosteric effects on microtubule structure, they could differentially impact cellular signalling pathways which ultimately dictate patient responses. Differences between paclitaxel and docetaxel were noted early in the clinical development of docetaxel^9^ and ixabepilone has activity in patients resistant to taxanes.^10^ More recently in the EMBRACE trial, eribulin provided a survival advantage compared to treatment of physician’s choice in patients who had failed to respond to a taxane and an anthracycline.^11^ Additionally, breast cancer cell lines also show different sensitivities to these drugs.^12^ Although microtubule targeting agents are often conceptually grouped together, these studies highlight the importance of understanding the differences among microtubule targeting agents that might underlie optimal utility of individual drugs of this class.

The TGF-β pathway is a master regulator of EMT due to its ability to activate multiple transcriptional pathways that ultimately coordinate to drive a cell towards a mesenchymal phenotype. In canonical TGF-β signalling, TGF-β binds the TGF-β receptor I, a serine/threonine receptor kinase, which transactivates TGF-β receptor II to both recruit and phosphorylate Smad2 and Smad3. Activated Smad2/3 translocates into the nucleus along microtubules in a dynein-dependent manner.^13^ Phosphorylated Smad2 and Smad3 require dynein-light chains km23-1 and km23-2, respectively, for their nuclear transport, and depletion of either of these two dynein light chains inhibits Smad2/3 transcriptional activity.^14–16^ In the nucleus, activated Smad2/3 complexes with Smad4 to regulate target gene expression, including *SNAI1*, *SNAI2*, *ZEB1*, and *TWIST1*, genes central to EMT.^17,18^

Eribulin has been shown to reverse EMT in triple-negative breast cancer (TNBC) cells, in a TNBC xenograft model^19^ and in patients.^20^ A 7-day exposure of cells to eribulin initiated EMT reversal that was defined by phenotypic changes, including loss of N-cadherin and expression of E-cadherin together with suppression of Snail.^19^ Eribulin also initiated EMT reversal in a TGF-β-induced model of EMT.^19^ However, the underlying molecular mechanisms describing how eribulin-mediated microtubule disruption can ultimately lead to EMT-reversal remains unidentified. We hypothesised that microtubule disruption would inhibit TGF-β-induced expression of the breast cancer EMT-driving transcription factors Snail (*SNAI1*) and Slug (*SNAI2*) because a key mediator of their transcription, Smad2/3, is trafficked to the nucleus along microtubules.^21^

## Methods

### Cell lines and reagents

BT-549 cells were obtained from the Lombardi Comprehensive Cancer Center, Georgetown University, Washington, DC. SUM185PE cells were purchased from BioIVT (Westbury, NY) and CAL-51 cells were purchased from Creative Bioarray (Shirley, NY). All other cell lines were purchased from ATCC (Manassas, VA). Cell lines were validated by Genetica (Burlington, NC). Cells were maintained in the following growth media: MDA-MB-231, SUM185PE, MDA-MB-453 in IMEM; BT-549, HCC1937, MCF-7, T47D, MDA-MB-468, HCC1806, CAL-51 in RPMI, and Hs578T in DMEM. Media were supplemented with 10% FBS and 50 μg/mL gentamycin, and cells were grown at 37 °C with 5% CO_2_. Replete conditions refer to cells grown in complete media whereas serum-starved conditions denote cells placed in media supplemented only with gentamycin. Paclitaxel (Sigma Aldrich, St Louis, MO), ixabepilone (LC Labs, Woburn, MA), vinorelbine (AdooQ Biosciences, Irvine, CA), docetaxel (Sigma Aldrich) and eribulin (Eisai Inc., Woodcliff Lake, NJ) were reconstituted in DMSO and stored at −20 °C. TGF-β was purchased from R&D Systems (Minneapolis, MN, Cat. 240-B-002) and reconstituted per manufacture’s recommendation in sterile 4 mM HCl in H_2_O containing 0.1% bovine serum albumin. TGF-β used was used at 2 ng/mL unless otherwise noted.

### Western blotting

Cells were lysed with cell extraction buffer (Life Technologies, Carlsbad, CA,) supplemented with protease inhibitors (Sigma Aldrich, Cat. P2714) and PMSF (Sigma Aldrich). Equal amounts of proteins were resolved by PAGE and evaluated by immunoblotting. The list of antibodies is provided in Supplementary Table 1. REVERT™ (LI-COR Biosciences, Lincoln, NE) protein stain was used to measure total protein on a membrane and the Odessey® Fc Imager (LI-COR Biosciences) was used to image all western blots.

### Indirect immunofluorescence

Cells were plated on coverslips and fixed with 4% paraformaldehyde (Electron Microscopy Sciences, Hatfield, PA). Cells were permeabilized using 0.5% Triton-X in PBS and blocked using 10% bovine calf serum in PBS, followed by immunostaining. Immunofluorescence images were obtained using a Nikon Eclipse 80i fluorescence microscope using NIS elements software to deconvolve and focus stacked images or the Operetta™ high content imager (PerkinElmer, Waltham, MA) as indicated.

For Operetta™ imaging, cells were plated in 96-well cell-carrier plates (PerkinElmer) and imaged in a single focal plane at ×20 magnification. Using Columbus™ (PerkinElmer) software, the protein intensity in the cytoplasm and nucleus were quantified. The nucleus and cytoplasm were defined by DAPI staining and CellMask™ Blue (Thermo Fisher Scientific) staining, respectively. The percentage of protein in the nucleus was calculated as the ratio of signal intensity present in the nucleus divided by the signal intensity present in the entire cell. The percentage of protein present in the nucleus was calculated for every cell and median values reported per well. To compare treatment conditions, the average of these median values was calculated for multiple wells within each independent experiment.

### qRealTime-PCR

RNA was extracted using the RNeasy mini kit (Qiagen, Germantown, MD) and purified using the RNase-Free DNase kit (Qiagen). RNA was reverse transcribed to cDNA using the High-capacity cDNA reverse transcription kit (Thermo Fisher Scientific) in the T100™ thermal cycler (Bio Rad, Hercules, CA). SYBR green chemistry (SYBR green master mix, Applied Biosystems, Foster City, CA) was used to evaluate transcript abundance by the CFX Connect™ real-time PCR detection system (Bio Rad). mRNA fold change was calculated using the 2^-ΔΔCt^ method,^22^ where GAPDH was used as the control gene. For all TGF-β-stimulation experiments, the fold-change of the mRNA of interest was set to 1 in the vehicle-unstimulated sample and all other treatments were reported relative to this sample. The list of primers is provided in Supplementary Table 2.

### Chromatin immunoprecipitation

Pierce™ Magnetic ChIP kit (Thermo Fisher Scientific, Cat. 26157) was used for chromatin immunoprecipitation. Nuclear lysate was pre-cleared with normal IgG followed by protein A/G beads. Equivalent amounts of nuclear lysate were used for Smad4 immunoprecipitation and IgG immunoprecipitation, while 10% was saved as input. Smad4 and IgG bound DNA were evaluated for *SNAI1* and *ID1* promoter regions using qPCR, and data are reported relative to input for each treatment condition.

### siRNA transfection

Cells were transfected using Lipofectamine RNAiMAX (Thermo Fisher Scientific Cat. 13778150) as per the manufacturer’s recommendation. Pools of pre-designed siRNAs targeted to *SMAD2* (SASI_Hs02_0030429, SASI_Hs02_00304296), *SMAD3* (SASI_Hs01_00208931, SASI_Hs02_00340511), *SMAD4* (SASI_Hs01_00207793, SASI_Hs01_00207794) and *JUN* (SASI_Hs02_00333461, SASI_Hs01_00150279), were purchased from Sigma Aldrich. Mock-transfection consisted of transfection reagent only. For the Smad4 and c-Jun knock-down, the two sets of siRNAs were used at equimolar concentrations for a final concentration of 100 nM, while the Smad2/Smad3 knock-down used equimolar concentrations of the siRNAs for a final siRNA pool concentration of 200 nM. All siRNA experiments were terminated after 72 h of transfection. The 72 h time period included serum-starvation, drug treatment and TGF-β stimulation.

### Transient transfection

pBabe-puro-Smad4-Flag was a gift from Sam Thiagalingam (Addgene plasmid #37041).^23^ Plasmid was purified using the Qiagen QIAprep® spin miniprep kit and 1 µg of plasmid was used to transfect a single well of a 6-well dish containing MDA-MB-468 cells using the Lipofectamine 3000 reagent (ThermoFisher) as per manufacturer’s instructions. Selection with 1 µg/mL of puromycin (InvivoGen, San Diego, CA) was initiated within 24 h of transfection. The transfection experiments were terminated within 72 h of transfection. The 72 h time period included puromycin treatment, serum-starvation and drug treatment. The results from this experiment were compared to un-transfected and drug-treated MDA-MB-468 cells shown in Fig. 5b.

### TCGA data analysis

cBioPortal was used to analyse the METABRIC dataset^24,25^ consisting of genomic landscape and gene expression data of 2509 samples. *SMAD4* and *SNAI2* were used as query genes, and their mapped mutations, copy number alterations, and deletions were analysed. The changes in mRNA expression were assessed using a threshold Z-score of ± 2.0. The Kaplan–Meier curves were generated by the cBioPortal software, where overall survival of patients with tumours harbouring alterations in query genes were compared to patients with tumours not containing any alterations in the query genes. The Logrank test was conducted to compare survival distribution of the two sample subsets.

## Results

### Effect of microtubule targeting agents on TGF-β-mediated Snail expression

**Fig. 1:**
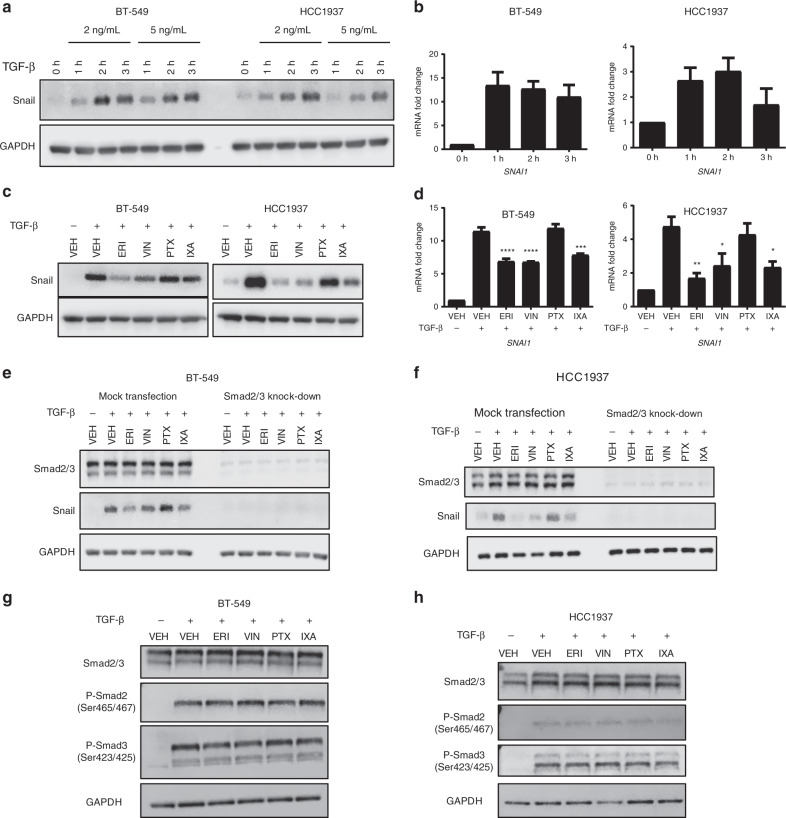
TGF-β causes rapid induction of Snail, which is differentially inhibited by microtubule targeting agents. **a** Western blot analysis of lysates from BT-549 and HCC1937 cells that were serum-starved for 18 h then stimulated with TGF-β at the indicated times and concentrations. Immunoblots were probed for Snail and GAPDH. **b** mRNA expression analysis of *SNAI1* in BT-549 and HCC1937 cells. Cells were serum-starved for 18 h then stimulated with 2 ng/mL of TGF-β for indicated times. Data are an average of two independent experiments (±SEM). **c** Western blot analysis of Snail and GAPH from whole-cell lysates from BT-549 and HCC1937 cells that were serum-starved for 18 h, then pre-treated with microtubule targeting agents for 2 h followed by a 3 h stimulation with TGF-β. **d** mRNA analysis of *SNAI1* transcript in cells that were serum-starved for 18 h, pre-treated with microtubule targeting agents for 2 h, then stimulated with TGF-β for either 1 h (BT-549 cells, *N* = 3) or 2 h (HCC1937 cells, *N* = 4). Data are the average of independent experiments (± SEM). A one-way ANOVA with Dunnett’s post-hoc test was used to determine statistical significance as compared to TGF-β-stimulated vehicle control cells (****p < 0.0001, ***p < 0.001, **p < 0.01, *p < 0.05). **e**, **f**. Smad2/3 proteins were transiently knocked-down by siRNA in BT-549 and HCC1937 cells after which cells were serum-starved for 18 h, pre-treated with microtubule targeting agents for 2 h, and then stimulated with TGF-β for 3 h. Whole-cell lysates were subject to immunoblotting and probed for Smad2/3, Snail, and GAPDH. **g**, **h** Western blot analysis of whole-cell lysates from cells serum-starved for 18 h, pre-treated with microtubule targeting agents for 2 h, then stimulated with TGF-β for either 45 min (BT-549) or 1 h (HCC1937). Immunoblots were probed for total Smad2/3, their phosphorylated forms, and GAPDH

The ability of eribulin to rapidly interrupt TGF-β-mediated expression of EMT-promoting transcription factors was evaluated in breast cancer cell lines and compared to the effects of other microtubule targeting agents. The goal was to evaluate the early, initial effects of these drugs on key drivers of EMT in breast cancer that could explain the ability of eribulin to reverse EMT in patients and preclinical models.^19,20^ The experimental design utilised a 2 h pre-treatment of cells with microtubule targeting agents at concentrations that are clinically relevant and that cause maximal microtubule disruption.^8^ A 2 ng/mL concentration of TGF-β was sufficient to induce robust Snail protein (Fig. 1a) and *SNAI1* transcript expression (Fig. 1b and Supplementary Fig. S1a, b) within 2–3 h in serum-starved TNBC cells. The effects of a 2 h pre-treatment of cells with vehicle, eribulin (ERI), vinorelbine (VIN), paclitaxel (PTX), or ixabepilone (IXA) on TGF-β-induced Snail expression were then evaluated. The results show that the microtubule destabilizers eribulin and vinorelbine inhibited TGF-β-induced Snail expression in BT-549, HCC1937, MDA-MB-231, and Hs578T cells (Fig. 1c and Supplementary Fig. S1c, d). The microtubule stabilizer ixabepilone also attenuated Snail expression in three of the four cell lines. In contrast, paclitaxel had no effect on TGF-β-induced Snail expression in any of these cell lines (Fig. 1d and Supplementary Fig. S1e, f). Similar effects were observed in replete medium without exogenous TGF-β, albeit with a lower signal than was observed with acute pathway stimulation (Supplementary Fig. S2). To determine whether the effects of the microtubule targeted drugs on Snail were mediated by transcriptional changes we evaluated the Snail transcript, *SNAI1*. Eribulin, vinorelbine and ixabepilone caused significant inhibition of *SNAI1* in BT-459 and HCC1937 cells (Fig. 1d) but only eribulin and vinorelbine reduced *SNAI1* transcript in MDA-MB-231 and Hs578T cells (Supplementary Fig. S1e, f). These results demonstrate that a 2 h pre-treatment with eribulin, vinorelbine, and in some cases ixabepilone, is sufficient to inhibit the transcriptional activation of the TGF-β target gene *SNAI1*, resulting in reduced Snail expression.

### Effect of microtubule targeting agents on Smad2/3 nuclear localisation

To address how some microtubule targeting agents inhibit *SNAI1* transcription, their effects on upstream events were evaluated. Smad2/3/4 proteins are canonical TGF-β pathway mediators and part of the transcriptional machinery responsible for *SNAI1* expression. siRNA mediated depletion of Smad2/3 was used to evaluate whether the effects of microtubule targeting agents on *SNAI1* were mediated by this canonical pathway. Consistent with the results presented in Fig. 1, eribulin, vinorelbine, and ixabepilone, but not paclitaxel, inhibited TGF-β-induced Snail expression in mock-transfected cells (Fig. 1e, f). Smad2/3 depletion completely attenuated TGF-β-mediated Snail expression in BT-549 and HCC1937 cells (Fig. 1e, f) (upper band – Smad3, lower band – Smad2). These results demonstrate that Smad2/3 is required for Snail expression and that this signalling occurs through the canonical TGF-β pathway.

An initial step in the execution of the canonical pathway is the phosphorylation and activation of Smad2/3 following TGF-β stimulation, suggesting that this could be a point of pathway inhibition by microtubule targeting agents. However, the results (Fig. 1g, h) show that microtubule targeting agents do not affect Smad2/3 phosphorylation levels following TGF-β stimulation suggesting that the ability of microtubule targeting agents to inhibit Snail expression is not due to changes in the initial activation (phosphorylation) of Smad2/3.

**Fig. 2:**
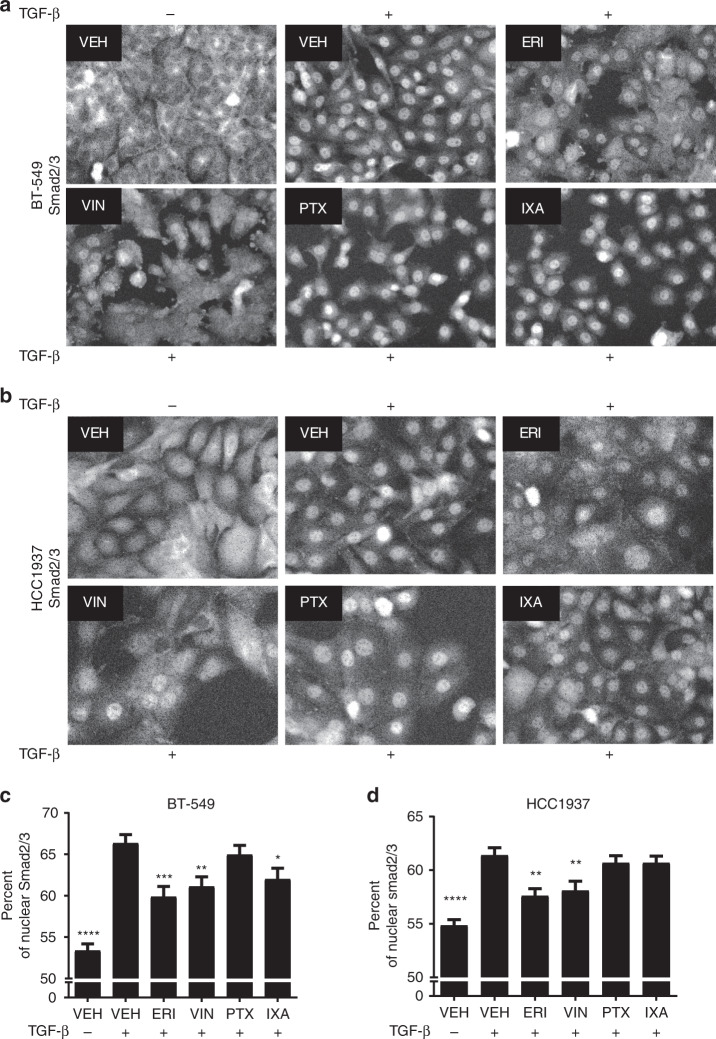
Microtubule targeting agents differentially impair the TGF-β-mediated nuclear accumulation of Smad2/3. **a**, **b** Smad2/3 localisation was evaluated by immunofluorescence in BT-549 and HCC1937 cells that were serum-starved for 18 h, pre-treated with microtubule targeting agents for 2 h, and stimulated with TGF-β for either 45 min (BT-549) or 1 h (HCC1937). Images were obtained using the Operetta™ high content imager and are representative of four independent experiments. **c**, **d** The percentage of Smad2/3 present in the nucleus of BT-549 and HCC1937 cells was quantified. Data are the average of four individual experiments, each with four replicates (mean ± SEM, *N* = 16). One-way ANOVA with Dunnett’s post-hoc test was used to determine statistical significance as compared to TGF-β-stimulated vehicle controls. (****p < 0.0001, ***p < 0.001, **p < 0.01, *p < 0.05)

After activation, Smad2/3 proteins translocate from the cytoplasm to the nucleus to initiate transcription. Since cytosolic Smad2/3 traffics to the nucleus along microtubules in a dynein-dependent manner^14–16^ and microtubule targeting agents have been shown to inhibit nuclear transport of other transcription factors,^26^ the effects of microtubule targeting agents on the TGF-β-induced nuclear accumulation of Smad2/3 were evaluated. TGF-β initiated a robust time-dependent increase in nuclear Smad2/3 in BT-549 and HCC1937 cells within 1 h (Supplementary Fig. S3). However, in cells pre-treated with eribulin or vinorelbine, the TGF-β-mediated nuclear localisation of Smad2/3 was significantly inhibited as compared to TGF-β stimulated controls, with a more dispersed cytoplasmic localisation of Smad2/3 observed in both cell lines (Fig. 2 and Supplementary Fig. S4). In contrast, paclitaxel had no effect on Smad2/3 localisation in either cell line, while ixabepilone reduced Smad2/3 nuclear localisation in BT-549 but not HCC1937 cells. The eribulin and vinorelbine-mediated inhibition of nuclear Smad2/3 localisation is consistent with their ability to attenuate *SNAI1* transcript and Snail protein expression.

### Effects of microtubule targeting agents on Smad4-dependent Snail expression

**Fig. 3:**
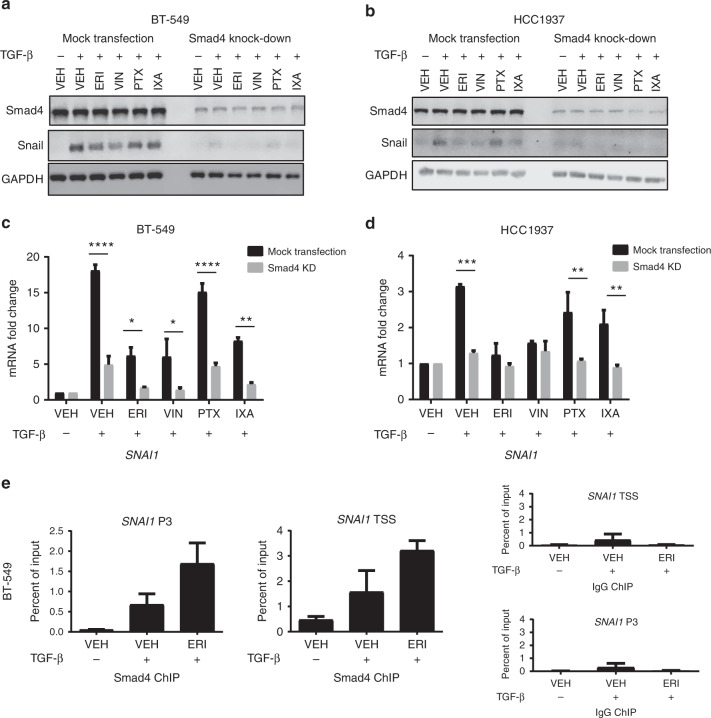
Smad4 is required for Snail expression. **a**, **b** Smad4 was knocked-down in BT-549 and HCC1937 cells by siRNA after which cells were serum-starved for 18 h, pre-treated with microtubule targeting agents for 2 h, and stimulated with TGF-β for 3 h. Cell lysates were subject to immunoblotting and probed for Smad4, Snail, and GAPDH. **c**, **d** qRT-PCR analysis of *SNAI1* transcript after siRNA-mediated knock-down of Smad4. Cells were serum-starved for 18 h, pre-treated with microtubule targeting agents for 2 h, and stimulated with TGF-β for 1 h (BT-549) or 2 h (HCC1937). Data are the average of two independent experiments (±SEM). Statistical significance of Smad4 knock-down for each condition was determined using a two-way ANOVA with Holm-Sidak’s multiple comparison test. (****p < 0.0001, ***p < 0.001, **p < 0.01, *p < 0.05) **e** ChIP-qPCR of two distinct *SNAI1* promoter regions. After 18 h of serum-starvation, BT-549 cells were pre-treated with either vehicle or eribulin for 2 h followed by stimulation with TGF-β for 1 h. Chromatin immunoprecipitation of *SNAI1* P3 and *SNAI1* TSS promoter sequences with Smad4 or control IgG are shown. Data are the mean ± SEM (*N* = 2) and reported as percentage of input chromatin for each sequence

In addition to Smad2/3, Smad4 is an integral part of the transcription machinery required to regulate *SNAI1* expression. The effects of Smad4 depletion on TGF-β-induced Snail expression were evaluated. Consistent with Smad2/3 knockdown, Smad4 depletion inhibited TGF-β-mediated Snail expression (Fig. 3a, b and Supplementary Fig. S5a, c) and *SNAI1* transcript (Fig. 3c, d and Supplementary Fig. S5b, d) in all four cell lines under all treatment conditions. The direct binding of Smad4 to the *SNAI1* promoter was evaluated by ChIP-qPCR. Enrichment of Smad4 at two distinct Smad-binding elements of the *SNAI1* promoter was observed with TGF-β stimulation of vehicle-treated cells (Fig. 3e). Interestingly, Smad4 enrichment at the *SNAI1* promoter was even higher in eribulin-treated cells when compared to vehicle (Fig. 3e), even though *SNAI1* expression was inhibited by eribulin. The same trend of increased Smad4 binding with eribulin pre-treatment was also observed at the promotor of another Smad-dependent gene, *ID1* (Supplementary Fig. S6a). The specificity of the Smad4 antibody was confirmed by the lack of enrichment of these *SNAI1* and *ID1* promoter regions in MDA-MB-468 cells that do not express Smad4^27^ (Supplementary Fig. S6b). Together, these data show that in the presence of eribulin, Smad4 retains the ability to localise to the nucleus and bind the *SNAI1* promoter. However, the eribulin-mediated inhibition of Smad2/3 transport into the nucleus results in a lack of Smad2/3/4-dependent transcriptional activation, likely leading to an accumulation of Smad4 at the promoter region. Thus, Smad4 binding to the *SNAI1* promoter is necessary, but not sufficient to induce *SNAI1* transcription after TGF-β stimulation. This could explain why increased Smad4 recruitment to the *SNAI1* promoter is unable to overcome eribulin-mediated inhibition of nuclear Smad2/3 accumulation resulting in decreased Snail expression.

### Effects of microtubule targeting agents on Slug expression

**Fig. 4:**
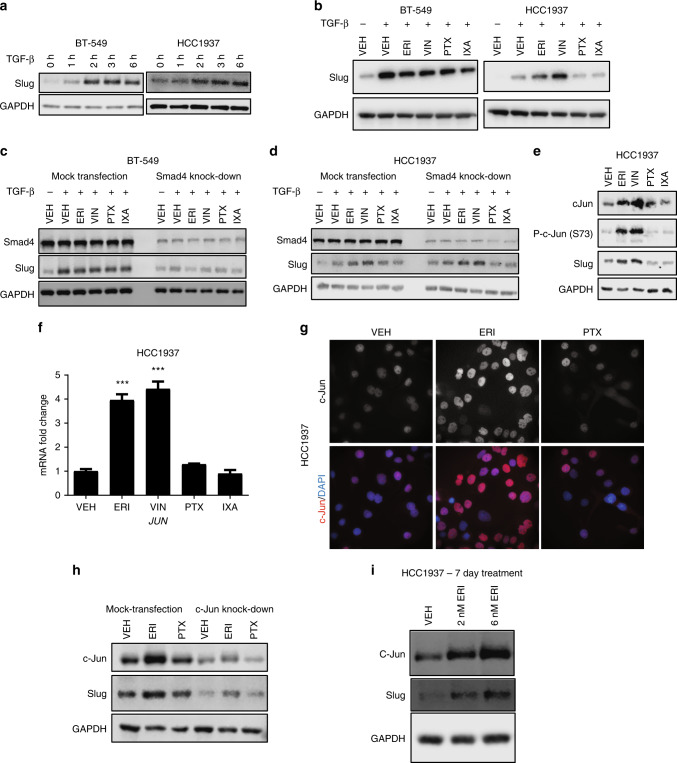
Microtubule targeting agents differentially impact Slug expression in breast cancer cell lines. **a** Western blot analysis of Slug from BT-549 and HCC1937 cells that were serum-starved for 18 h, and then stimulated with TGF-β for the indicated times. **b** Western blot analysis of Slug and GAPDH from whole-cell lysates from BT-549 and HCC1937 cells that were serum-starved for 18 h, pre-treated with microtubule targeting agents for 2 h followed by a 3 h stimulation with TGF-β. **c**, **d** Smad4 was transiently knocked-down by siRNA in BT-549 and HCC1937 cells. Cells were then serum-starved for 18 h, pre-treated with microtubule targeting agents for 2 h, then stimulated with TGF-β for 3 h. Cell lysates were subject to immunoblotting for Smad4, Slug, and GAPDH. **e** Western blot analysis of whole-cell lysates from HCC1937 cells that were serum-starved for 18 h and treated with microtubule targeting agents for 5 h. Immunoblots were probed for total and phosphorylated forms of c-Jun, Slug, and GAPDH. **f** mRNA analysis of *JUN* transcript in HCC1937 cells that were serum-starved for 18 h and then treated with microtubule targeting agents for 4 h (*N* = 2). Data are reported as mean ± SEM. Statistical significance compared to vehicle controls was determined using a one-way ANOVA with Dunnett’s post-hoc test (***p < 0.001). **g** HCC1937 cells were serum-starved for 18 h and then treated with microtubule targeting agents for 5 h. Immunofluorescence images of c-Jun and DAPI are shown. **h** c-Jun was transiently knocked-down in HCC1937 cells by siRNA. Cells were serum-starved for 18 h and then treated with microtubule targeting agents for 5 h. Cell lysates were subject to immunoblotting for c-Jun, Slug, and GAPDH. **i** Western blot analysis of whole-cell lysates from HCC1937 cells treated with vehicle or eribulin for 7 days. Immunoblots were probed for Slug, c-Jun, and GAPDH

In addition to Snail, Slug is another other important TGF-β responsive transcription factor that promotes EMT in breast cancer.^21,28^ Like Snail, Slug expression increased in a time-dependent manner after TGF-β stimulation with robust expression observed within 3 h in both BT-549 and HCC1937 cells (Fig. 4a). However, unlike what was observed for Snail, microtubule targeting agents had no appreciable effect on TGF-β-induced Slug expression in BT-549 cells (Fig. 4b). In contrast, in HCC1937 cells, eribulin and vinorelbine caused an unanticipated increase in Slug (Fig. 4b) and *SNAI2* transcript expression (Supplementary Fig. S7a). This effect was not seen with either paclitaxel or ixabepilone. In HCC1937 cells, Slug induction by eribulin or vinorelbine occurred independent of serum-starvation or TGF-β-stimulation (Supplementary Fig. S7b, c, d).

Previous studies showed that Slug expression can occur in a Smad4-independent manner.^29^ Therefore, the effects of Smad4 knock-down on microtubule destabilizer-mediated increases in Slug expression were evaluated. Depletion of Smad4 reduced TGF-β-induced Slug mRNA (Supplementary Fig. S8a) and protein expression (Fig. 4c) in BT-549 cells in the presence or absence of microtubule targeting agents, consistent with TGF-β-induced Slug expression being Smad4 dependent in this cell line. In contrast, in the HCC1937 cells, Smad4 depletion did not inhibit eribulin or vinorelbine-induced increases in Slug transcript (Supplementary Fig. S8b) or protein levels (Fig. 4d). Similar results were also seen with Smad2/3 depletion (Supplementary Fig. S8c, d), demonstrating that the eribulin- or vinorelbine-induced, TGF-β-independent expression of Slug in HCC1937 cells is Smad2/3/4 independent. Together, these data demonstrate that microtubule targeting agents can elicit different effects on Slug expression in distinct molecular contexts. This differs from the effects of microtubule targeting agents on Snail, which were generally consistent across all four TNBC cell lines evaluated.

### Role of c-Jun in microtubule destabilizer-mediated upregulation of Slug

We hypothesised that the mechanism of Smad-independent Slug upregulation by eribulin and vinorelbine in HCC1937 cells could be due to c-Jun, a transcription factor that positively regulates *SNAI2* expression^30^ and is upregulated after short-term treatment with the microtubule destabilizers nocodazole, colchicine, or vinblastine.^31,32^ The effects of microtubule targeting agents on c-Jun expression were evaluated in HCC1937 (Fig. 4e, f) and in BT-549 cells (Supplementary Fig. S9a). In each case, c-Jun and phospho-c-Jun were increased by eribulin and vinorelbine, but not paclitaxel or ixabepilone. Immunofluorescence experiments showed that the nuclear localisation of c-Jun was enhanced in eribulin-treated HCC1937 cells (Fig. 4g), where it can drive transcription of target genes, including *SNAI2*. Indeed, c-Jun depletion attenuated eribulin-mediated Slug upregulation in HCC1937 cells (Fig. 4h). These eribulin-mediated increases in c-Jun and Slug were also observed when HCC1937 cells were treated chronically (7 days) with low doses (1–6 nM) of eribulin in an experimental paradigm that has been shown to reverse EMT-associated phenotypes^19^ (Fig. 4i). In BT-549 cells, chronic treatment with low concentrations of eribulin led to increased c-Jun expression but no upregulation of Slug (Supplementary Fig. S9c), consistent with the response of these cells to eribulin in short-term experiments.

### Correlation of Smad4 expression levels with eribulin-mediated Slug upregulation

**Fig. 5:**
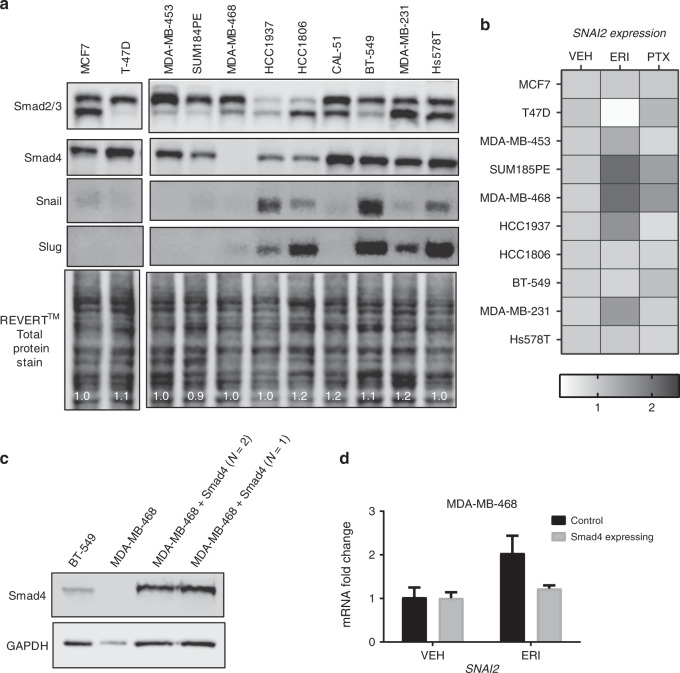
Eribulin-induced Slug expression correlates with endogenous Smad4 expression. **a** Western blot analysis of whole-cell lysates from 12 breast cancer cell lines. Immunoblots were probed for Smad2/3, Smad4, Snail and Slug. As a loading control, a total protein stain using the REVERT™ reagent is shown along with normalised lane quantifications. **b** Breast cancer cell lines were serum-starved for 18 h and treated with either vehicle, eribulin or paclitaxel for 4 h. qRT-PCR analysis was performed to evaluate the mRNA fold-change of *SNAI2*. Data are the average of two independent experiments in the form of a heat-map. **c** Whole-cell lysates from BT-549, MDA-MB-468 and Smad4-expressing MDA-MB-468 cells were evaluated for Smad4 and GAPDH by immunoblotting. **d** MDA-MB-468 and Smad4-expressing MDA-MB468 cells were serum-starved for 18 h and treated with either vehicle or eribulin for 4 h. *SNAI2* mRNA was analysed using qRT-PCR. Data are the average of two independent experiments (±SEM). Results were compared to un-transfected and drug-treated MDA-MB-468 cells shown in Fig. 5b, control

In efforts to understand why increased c-Jun expression resulted in increased Slug expression in HCC1937 but not BT549 cells, we noted that HCC1937 cells have lower Smad4 levels than BT-549 cells (Supplementary Fig. S9b). This led us to hypothesise that low Smad4 levels lead to c-Jun-dependent Slug expression in response to microtubule destabilization. To extend this observation, a panel of 12 breast cancer cell lines was used to evaluate the association between low Smad4 expression and microtubule-destabilizer-mediated Slug upregulation. The four cell lines expressing the lowest levels of Smad4 were SUM185PE, HCC1806, HCC1937, and MDA-MB-468 (Fig. 5a). Strikingly, HCC1937, SUM185PE, and MDA-MB-468 cells were the only models to elicit upregulation of *SNAI2* transcript after eribulin treatment (Fig. 5b and Supplementary Fig. S10a). Importantly, in the Smad4-null MDA-MB-468 cells, re-expression of Smad4 attenuated eribulin-mediated *SNAI2* upregulation (Fig. 5c). These data indicate that low Smad4 expression, particularly in more basal-like TNBC cells, is associated with microtubule destabilizer-mediated increases in Slug and that Smad4 expression is sufficient to attenuate this response. However, there are exceptions, for example, MDA-MB-231 cells respond to eribulin with an increase in *SNAI2* transcript even though they have high Smad4 levels, suggesting that other factors can be involved.

The METABRIC dataset^24,25^ was queried to evaluate the prognostic value of alterations in *SMAD4* and *SNAI2*. Using gene expression data from 2509 breast cancer patients followed for ~35 years, *SMAD4* or *SNAI2* mRNA expression alterations occur in 8 and 13% of patients, respectively. The overall low expression of Smad4 and high expression of Slug correlates with lower overall survival (Supplementary Fig. S11a). Additionally, when the same METABRIC dataset was evaluated and the subset of patients with genetic alterations (including mutations and copy-number alterations) in *SMAD4* (1.9% of all patients) and *SNAI2* (12% of all patients) were assessed, there was significantly lower overall survival in that subset of patients (Supplementary Fig. S11b).

Together, these findings indicate that a subset of breast cancer patients with low Smad4 expression might not be susceptible to the known EMT-reversing effects of eribulin, due to upregulation of the EMT-promoting transcription factor Slug. Consequently, we hypothesise that high Smad4 expression could serve as a potential biomarker of the ability of eribulin to reverse EMT in breast cancer patients.

## Discussion

**Fig. 6:**
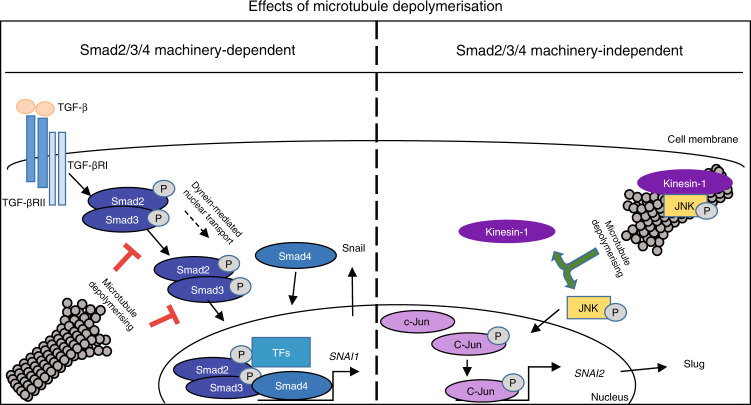
A diagram summarising the proposed effects of microtubule destabilization on expression of Snail and Slug, in a Smad2/3/4 molecular context-dependent manner

TGF-β initiates oncogenic signals that can transform cancer cells from an epithelial to a mesenchymal phenotype.^33,34^ We hypothesised that the ability of eribulin to reverse EMT in breast cancer cells, xenograft models, and circulating tumour cells in patients^19,20^ could be due to a rapid microtubule depolymerisation-mediated inhibition of TGF-β signalling. The effects of a panel of clinically relevant microtubule targeting agents on key breast cancer EMT-promoting transcription factors were investigated, with the goal of understanding the initial events that could link microtubule disruption to EMT reversal. Our results in a panel of TNBC cell lines show that the microtubule destabilizers, eribulin and vinorelbine, rapidly inhibit TGF-β-induced expression of Snail, a central transcriptional driver of EMT in breast cancer, providing a mechanism by which eribulin can downregulate the TGF-β/Smad signalling pathway.^19^ Attenuation of TGF-β signalling by eribulin and vinorelbine is associated with inhibition of Smad2/3 transport to the nucleus, a pathway that has previously been shown to be dynein and microtubule dependent.^14–16,35^ Inhibition of Smad2/3 nuclear accumulation diminished both *SNAI1* transcript and Snail protein expression after a 2 h pre-treatment with eribulin or vinorelbine demonstrating that microtubule depolymerisation rapidly disrupts canonical TGF-β signalling. While Smad2/3 nuclear transport was inhibited, the levels of Smad4 at the *SNAI1* promoter were elevated by eribulin, consistent with previous literature showing that the nuclear transport of Smad2/3 and Smad4 are independent.^27,36^ Together, these data show that nuclear delivery of Smad2/3, but not Smad4, is inhibited by microtubule destabilization and that Smad4 is necessary, but not sufficient, to initiate *SNAI1* transcription following TGF-β stimulation. This pathway is shown graphically in Fig. 6, left panel.

Our results showing that eribulin and vinorelbine inhibit Snail expression in each cell line evaluated suggests that this effect likely contributes to the ability of eribulin to reverse EMT in other experimental models.^19,20^ Consistent with the effects of the microtubule destabilizers, ixabepilone also inhibited TGF-β-induced *SNAI1* and Snail expression in BT-549 and HCC1937 cells, but not in MDA-MB-231 or Hs578T cells. In BT-549 cells the mechanism is likely mediated by attenuation of Smad2/3 nuclear transport, consistent with the effects of the microtubule destabilizers, however, in HCC1937 cells, ixabepilone did not inhibit nuclear Smad2/3 accumulation, suggesting another mechanism. Paclitaxel did not inhibit TGF-β-induced Snail expression in any of the four cell lines. The divergent effects of paclitaxel and ixabepilone demonstrate that significant differences exist even between these microtubule stabilizers. The ability of the microtubule destabilizers and, in some cell lines, ixabepilone, but not paclitaxel, to inhibit TGF-β-induced *SNAI1* and Snail expression could be important for their clinical efficacy, especially in breast cancer patients whose tumours have undergone EMT.

The EMT promoting transcription factors Snail and Slug are often considered as having similar effects; however, they regulate numerous non-overlapping genes.^28,37^ Genome-wide analysis of Snail and Slug promoter recruitment shows that Snail binds at ~8000 promoter regions while Slug binds at about 1500.^28^ Slug is expressed predominantly in the basal cells of the normal mammary duct while Snail is expressed in stromal fibroblasts,^28^ indicative of their distinct biological roles in normal mammary gland biology. Our data demonstrate that the effects of eribulin and vinorelbine on these two EMT-promoting transcription factors are distinct. While TGF-β-induced Snail expression was consistently inhibited by these drugs, TGF-β-mediated Slug expression was not inhibited by any of the microtubule targeting agents evaluated across a panel of molecularly diverse breast cancer cell lines. Surprisingly, in a subset of TNBC cell lines, eribulin and vinorelbine induced Slug expression in a TGF-β and Smad-independent manner. Slug expression is regulated by a variety of transcription factors, including c-Jun,^30,38^ and microtubule destabilizer-initiated c-Jun expression has been reported previously.^31,32^ However, the question of how microtubule depolymerisation increases c-Jun expression remains unanswered. c-Jun expression is regulated transcriptionally by c-Jun N-terminal kinase (JNK) and JNK is required for microtubule elongation through its direct binding to the motor protein kinesin-1.^39^ Additionally, pre-treatment of cells with a JNK inhibitor prevented microtubule-destabilizer-mediated c-Jun induction.^40^ We hypothesise that microtubule depolymerisation reduces the microtubule localisation of JNK, enabling it to activate c-Jun and this is shown in Fig. 6, right panel.

While the microtubule destabilizers increased c-Jun expression in all cell lines evaluated, Slug was preferentially upregulated by eribulin in cells with low Smad4 expression, suggesting that Slug is regulated by c-Jun when Smad4 is limited. Additionally, exogenous expression of Smad4 in a Smad4-null cell line attenuated the eribulin-mediated *SNAI2* upregulation, further indicating an important role of Smad4 in regulating this response. Smad4 is a predictive biomarker of poor prognosis in multiple cancers^41–46^ and the METABRIC data confirm that alterations in the *SMAD4* and *SNAI2* genes or their altered expression correlate with lower overall survival in breast cancer. Although this difference may be minor for prognosis in general, the specific role of Smad4 expression in response to microtubule targeting agents has not been systematically evaluated as a potential biomarker for response to these agents. We hypothesise that Smad4 expression would positively correlate with the known EMT-reversal in response to eribulin. In contrast, tumours with low Smad4 expression would not be expected to exhibit robust EMT reversal due to Slug upregulation is this molecular context.

Evidence is rapidly accumulating that the anticancer efficacy of microtubule targeting agents is not solely due to their antimitotic effects.^5,7,8^ The efficacy of these drugs warrants detailed investigations into their abilities to alter cell signalling pathways that could help explain their clinical efficacies in distinct molecular contexts. Differences between microtubule stabilizers and destabilizers and even among members of a single class with regard to inhibition of TGF-β signalling highlight the mechanistic distinctions among these drugs. Differences among microtubule stabilizers on microtubule structure and in patient responses are well documented,^47–50^ suggesting that distinct effects among drugs of this class on microtubule-associated signalling and trafficking are not unanticipated. The continued identification of signalling consequences that differ among microtubule targeting agents is critical to promote their utilisation in a more rational and targeted manner.^51^ Our data, together with other studies, demonstrate that microtubule targeting agents can disrupt key oncogenic signalling pathways and can do so differently. Our findings that eribulin and vinorelbine inhibit Snail expression in TNBC cell lines while inducing the expression of Slug specifically in cells with low Smad4 expression suggest that Smad4 should be evaluated as a predictive biomarker for the known EMT reversing effects of eribulin (summarised in cartoon, Fig. 6).

## Supplementary information

Supplemental Material File #1

## Data Availability

All data generated or analysed during this study are included in this article and its supplementary files. The METABRIC data set used is publicly available and was analysed using cBioPortal software.
