# Correction: Eribulin rapidly inhibits TGF-β-induced Snail expression and can induce Slug expression in a Smad4-dependent manner

**DOI:** 10.1038/s41416-020-01115-w

**Published:** 2020-10-15

**Authors:** Roma Kaul, April L. Risinger, Susan L. Mooberry

**Affiliations:** 1 Department of Pharmacology, University of Texas Health Science Center at San Antonio, San Antonio, TX USA; 2 Mays Cancer Center, University of Texas Health Science Center at San Antonio, San Antonio, TX USA

**Keywords:** Biomarkers, Molecular medicine

Correction to: *British Journal of Cancer* (2019) **121**, 611–621; 10.1038/s41416-019-0556-9, published online 4 September 2019

Since the publication of this paper, the authors have realised that the identification of the Smad2/3 antibody used was incorrect in Supplementary Table 1. The corrected identification of the Smad2/3 antibody is Cell Signaling Technologies, 8685 (D7G7). Supplementary Table 1 has been corrected accordingly. The authors would like to apologise for this error which does not alter the conclusions of the paper.

