# Supplementary material for: Eribulin rapidly inhibits TGF-β-induced Snail expression and can induce Slug expression in a Smad4-dependent manner
Source: Br J Cancer. 2019 Sep 4;121(7):611–21. doi: 10.1038/s41416-019-0556-9 (PMC6889360; doi:10.1038/s41416-019-0556-9)
Supplement: Supplementary file 1 — Supplemental Material File #1 [file 41416_2019_556_MOESM1_ESM.pdf]

Supplementary Figure 1

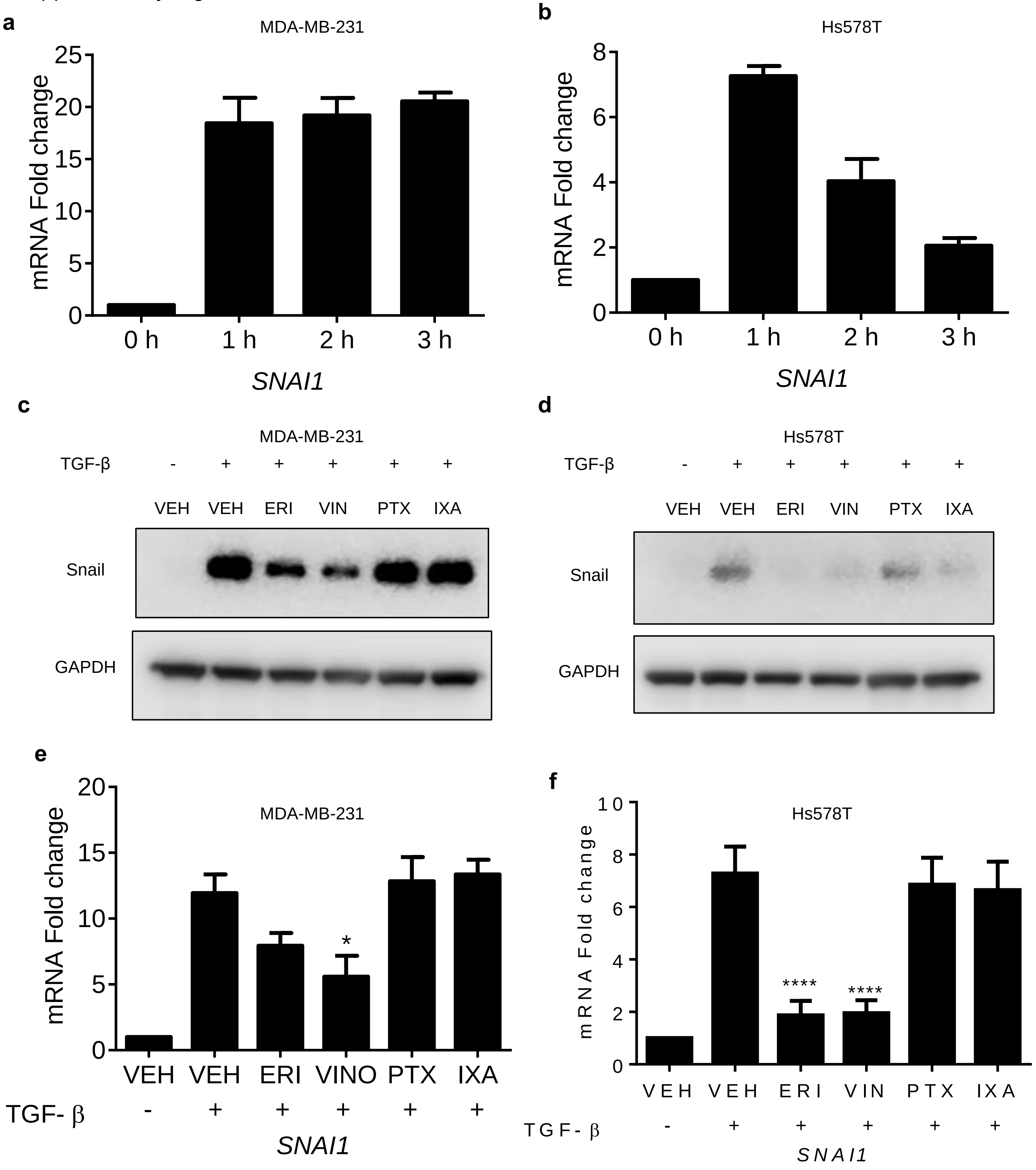

Supplementary Figure 2

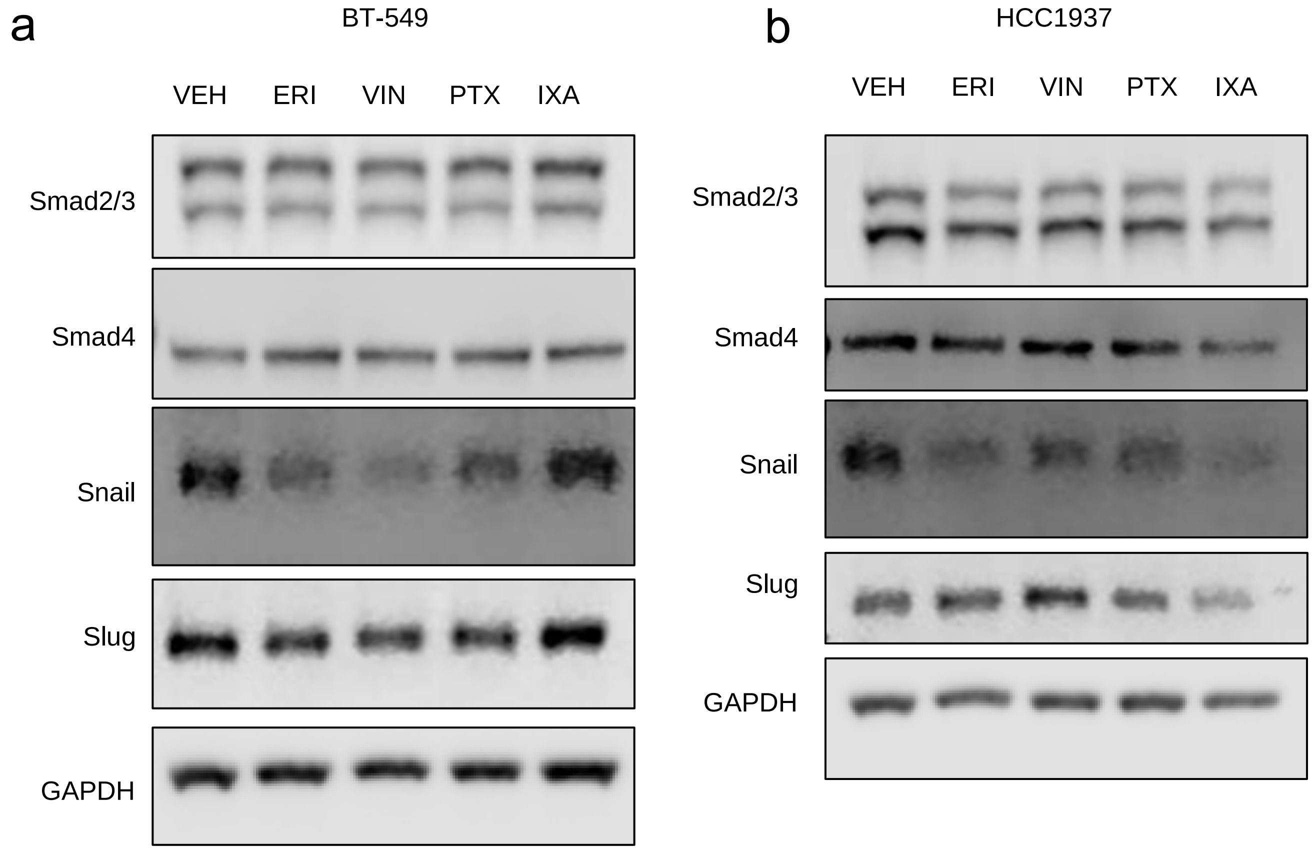

Supplementary Figure 3

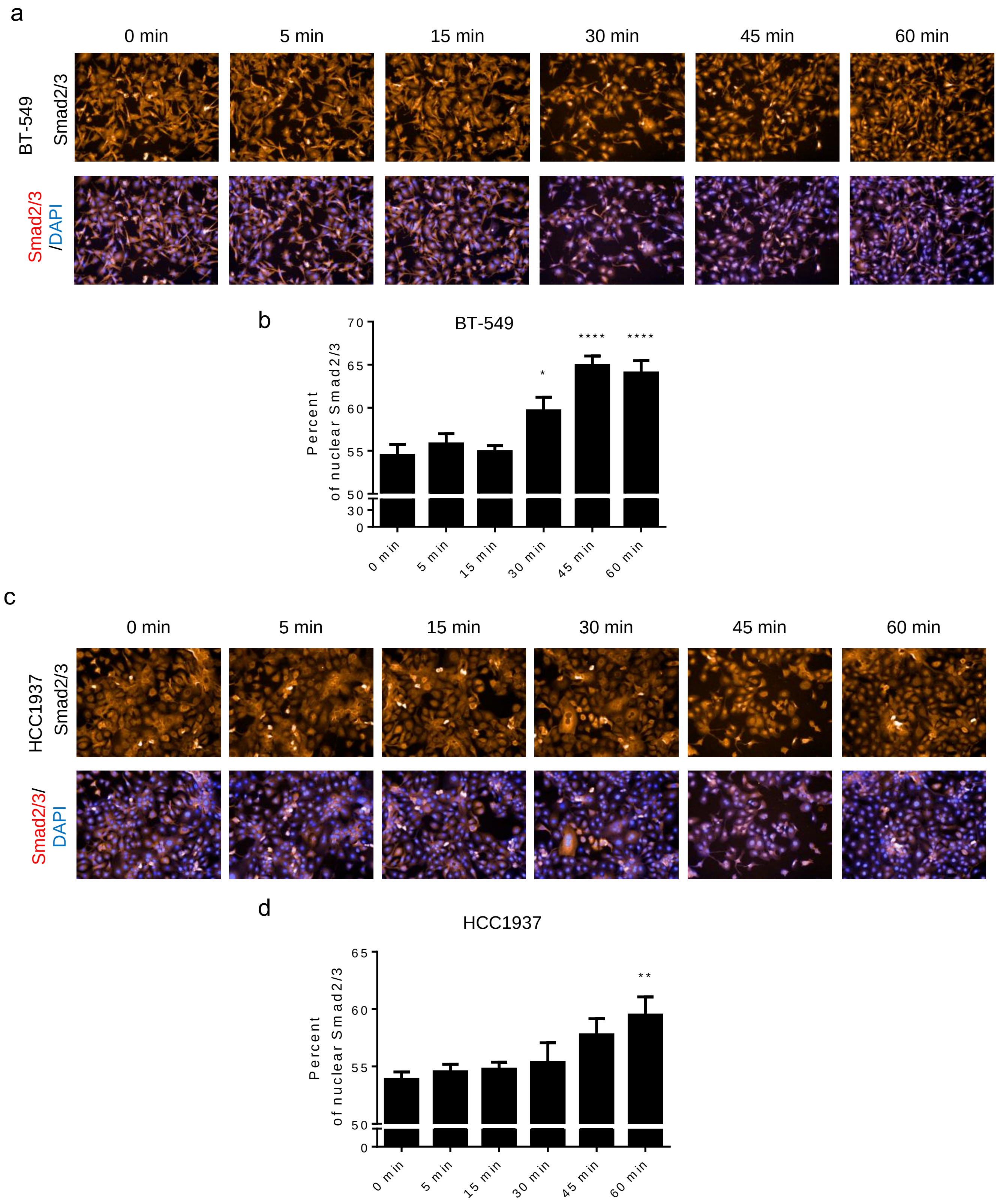

Supplementary Figure 4

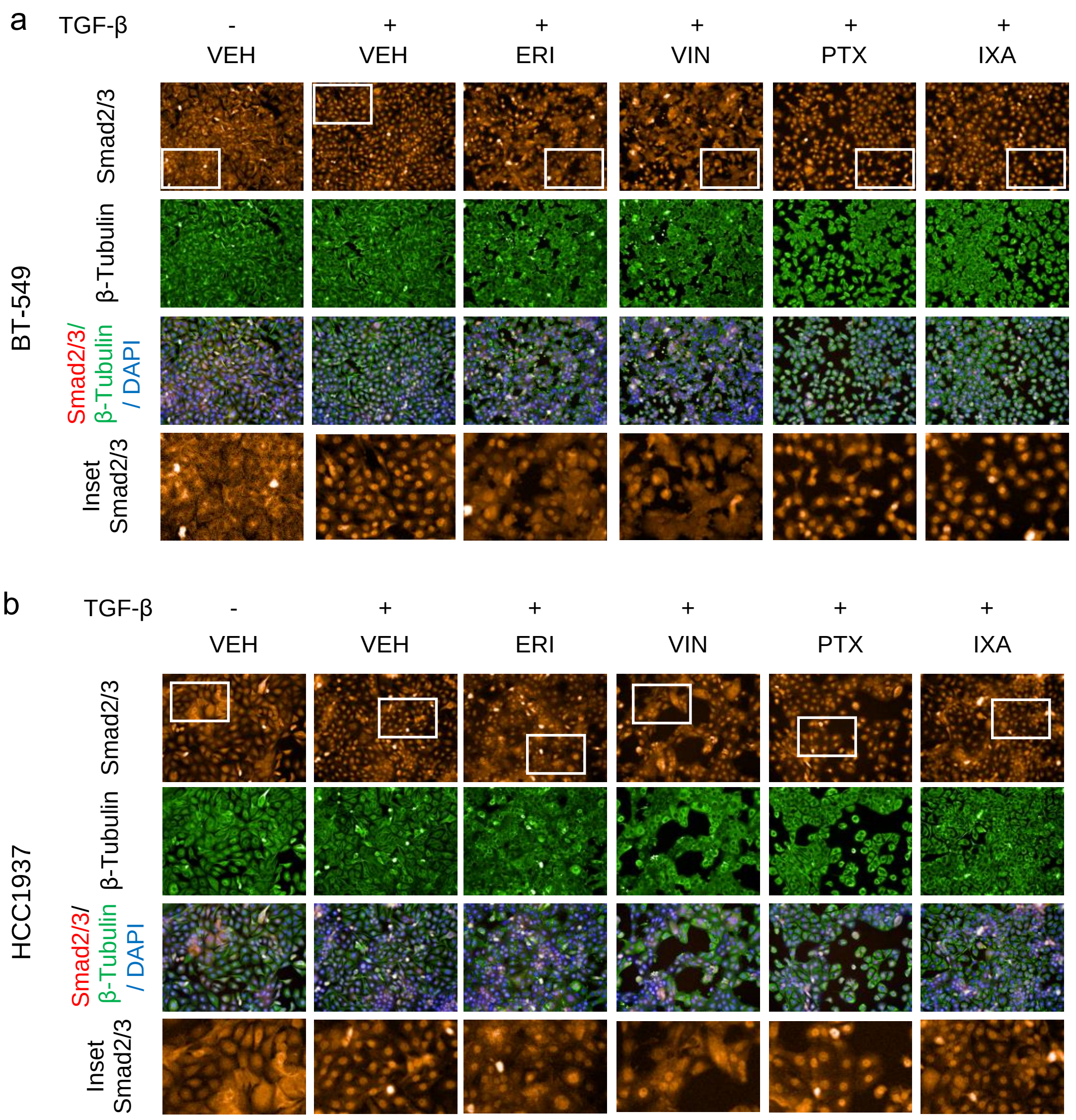

Supplementary Figure 5

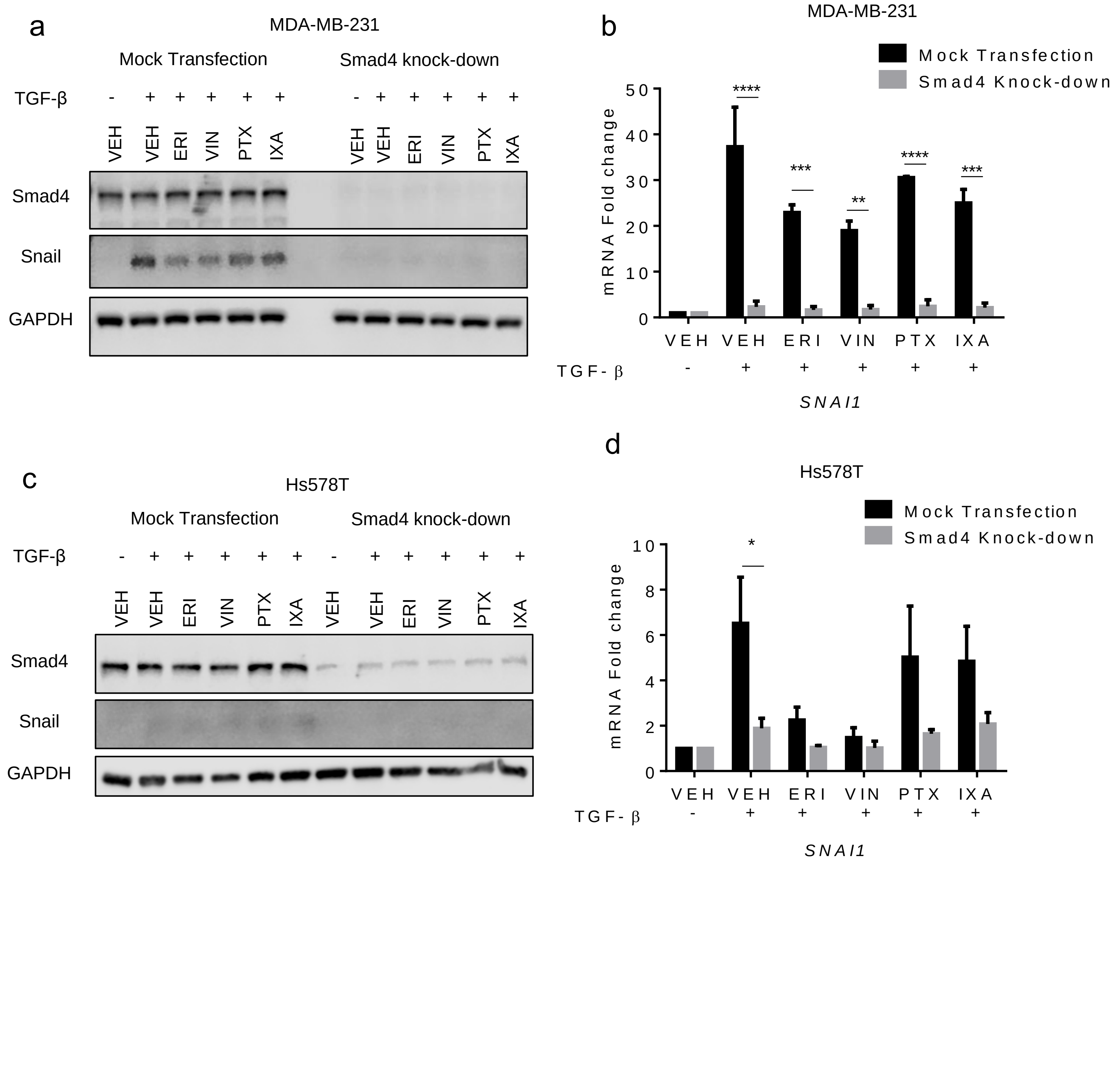

Supplementary Figure 6

a

BT-549

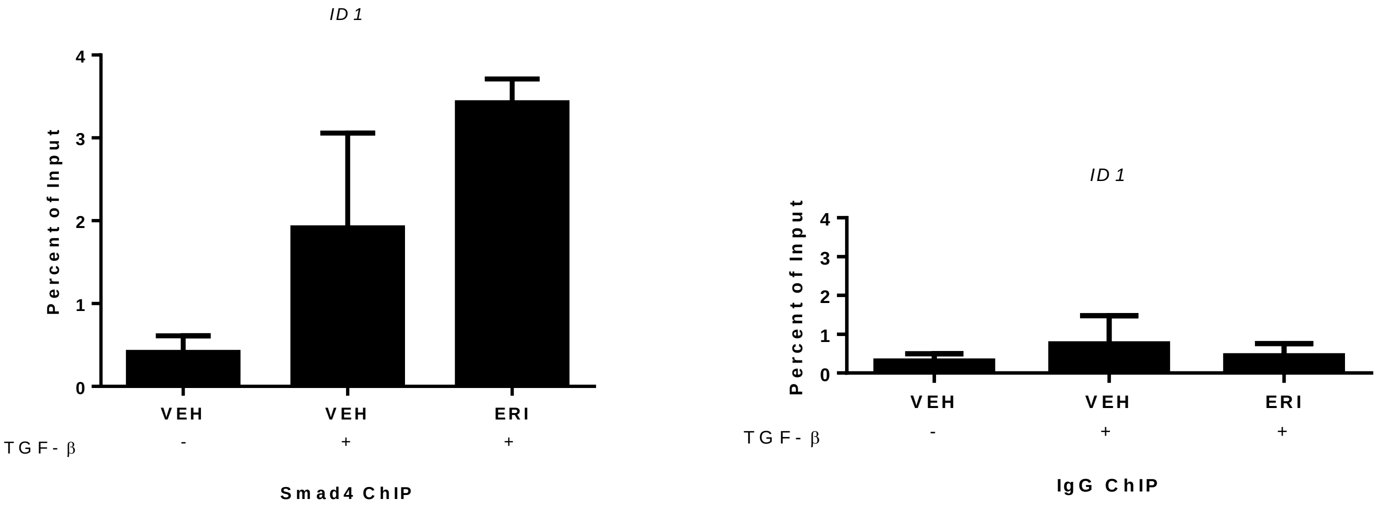

b

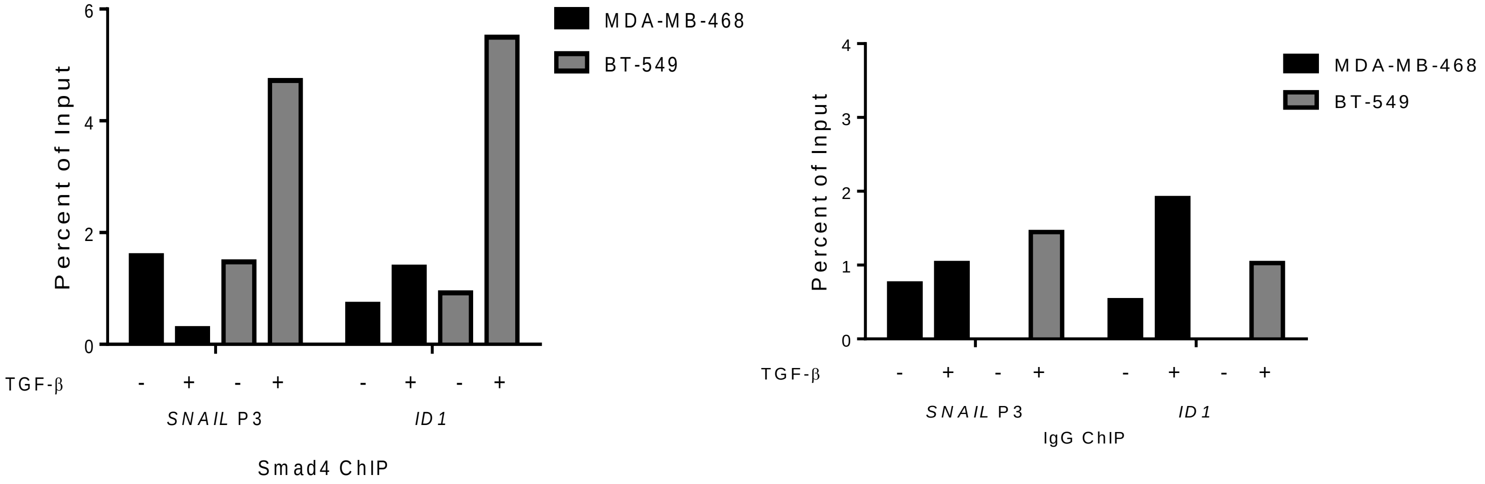

Supplementary Figure 7

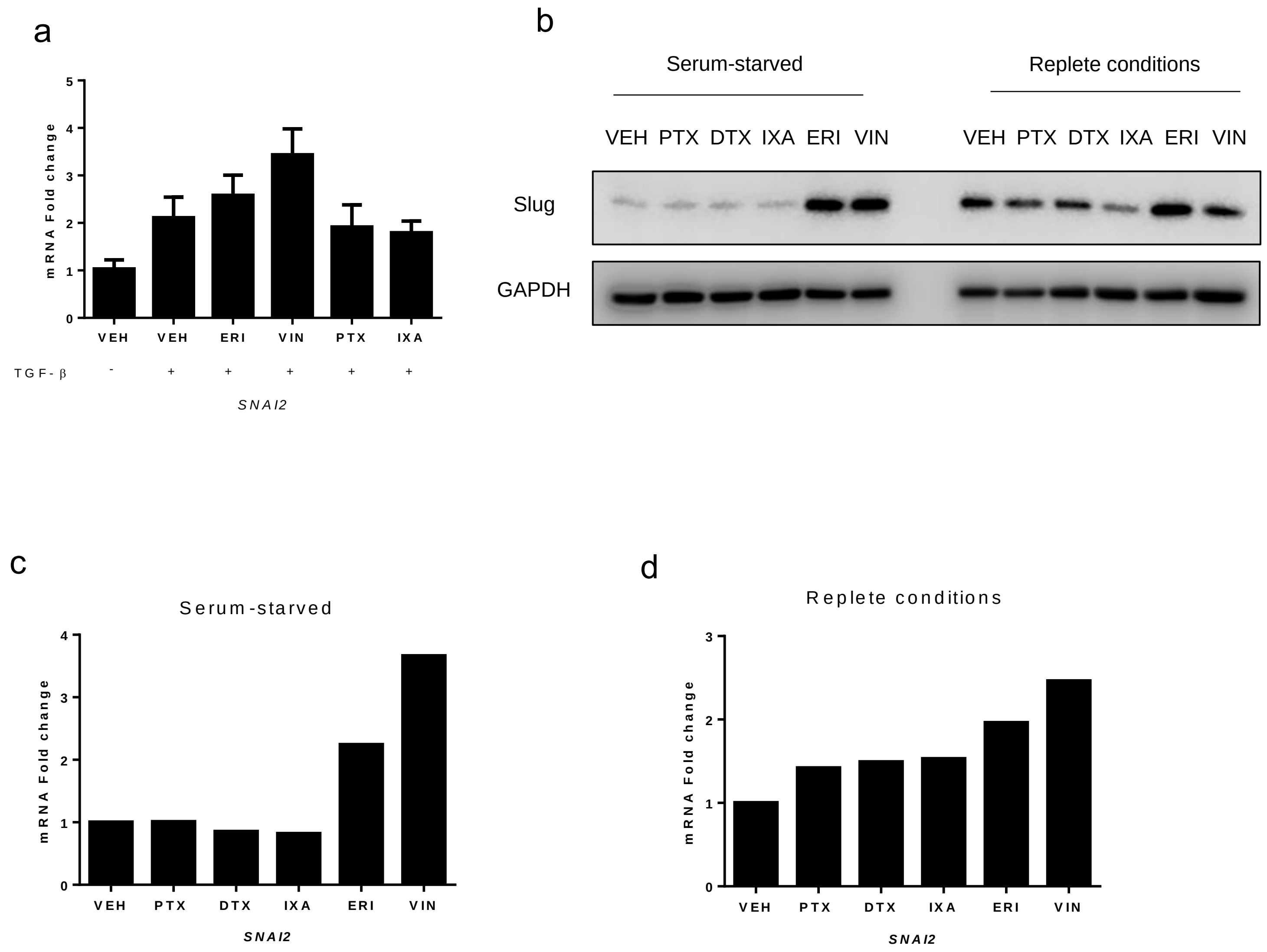

Supplementary Figure 8

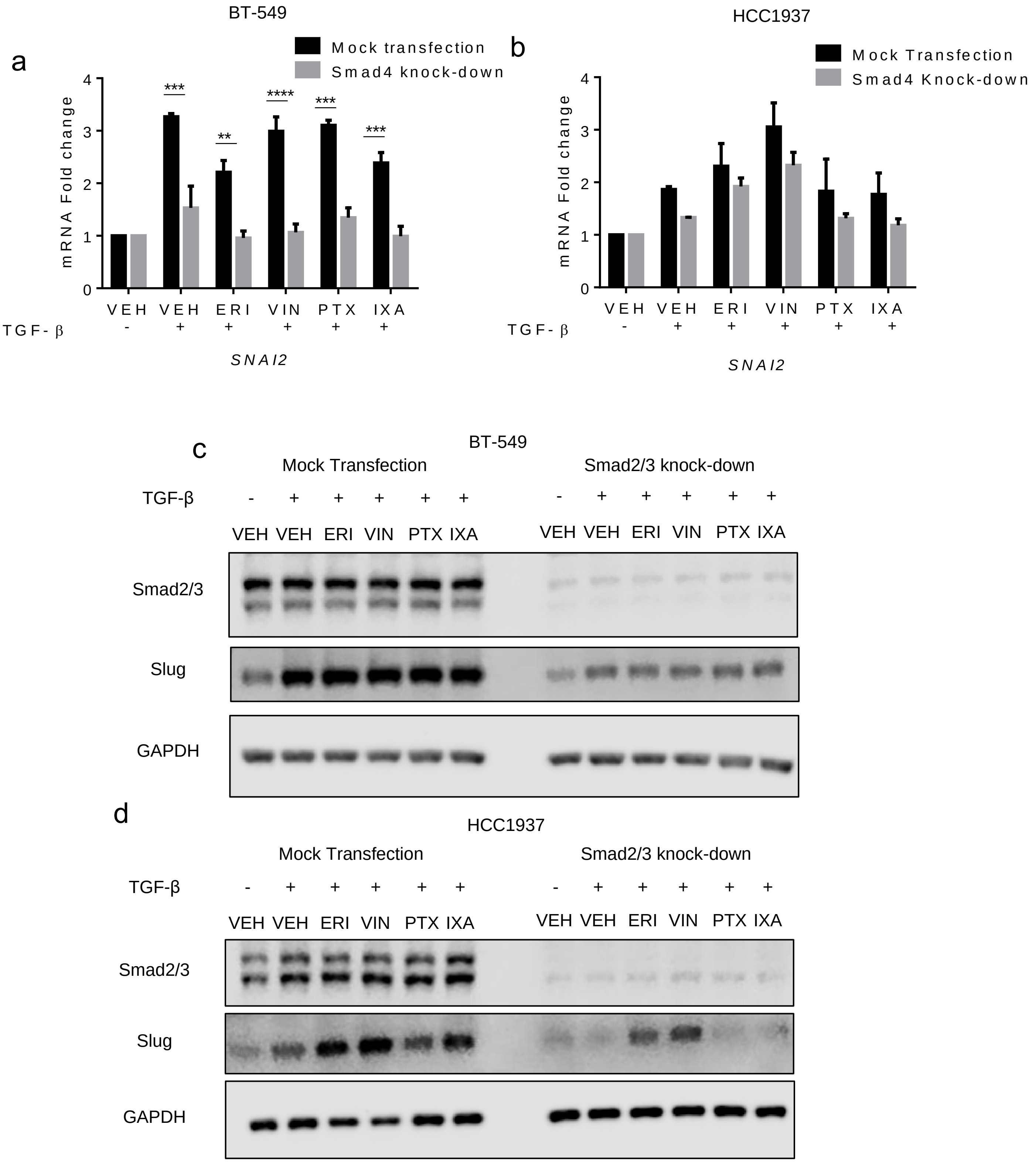

Supplementary Figure 9

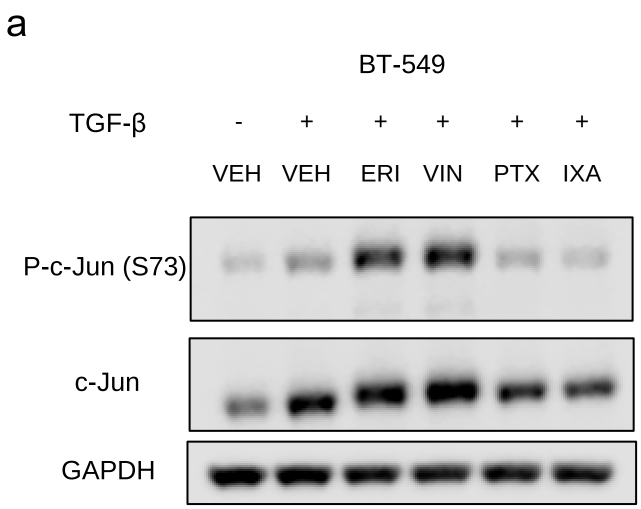

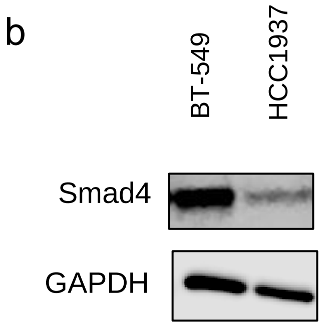

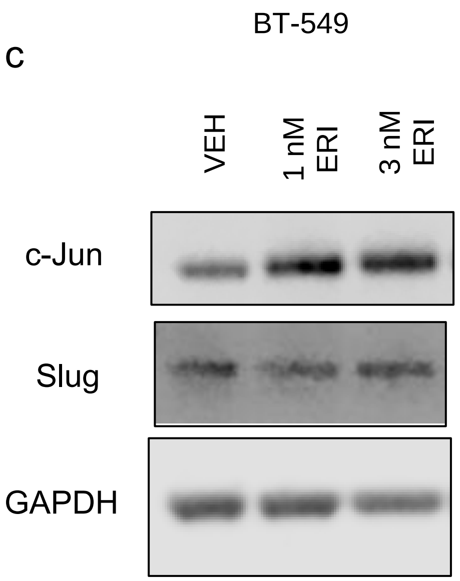

Supplementary Figure 10

a

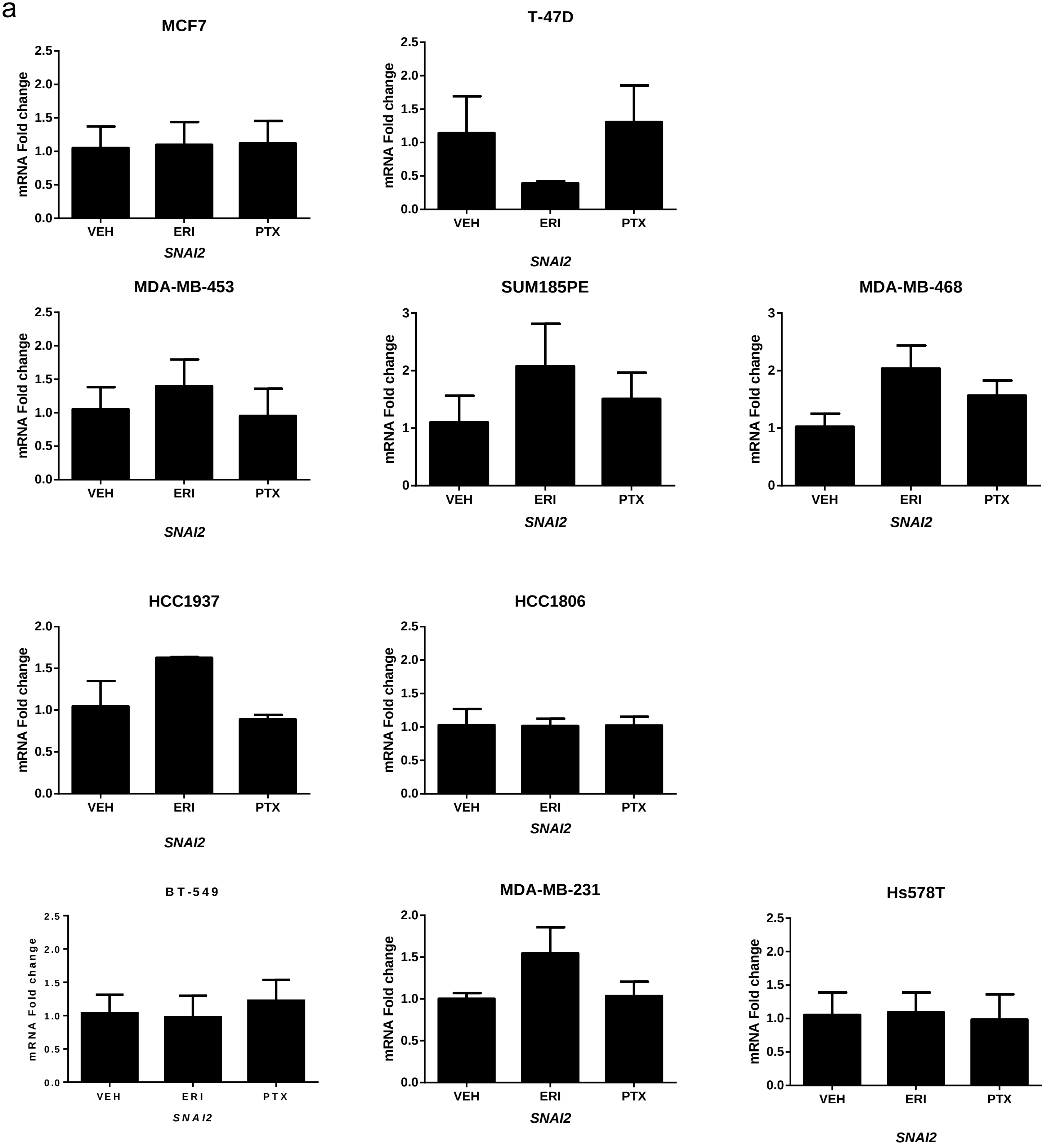

Supplementary Figure 11

a

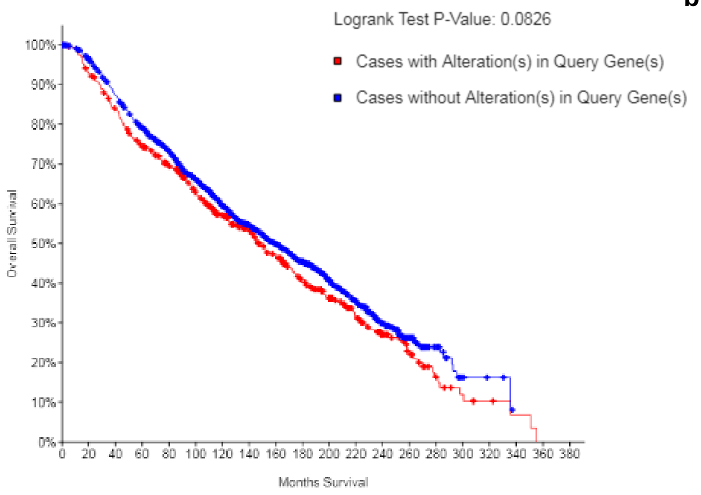

b

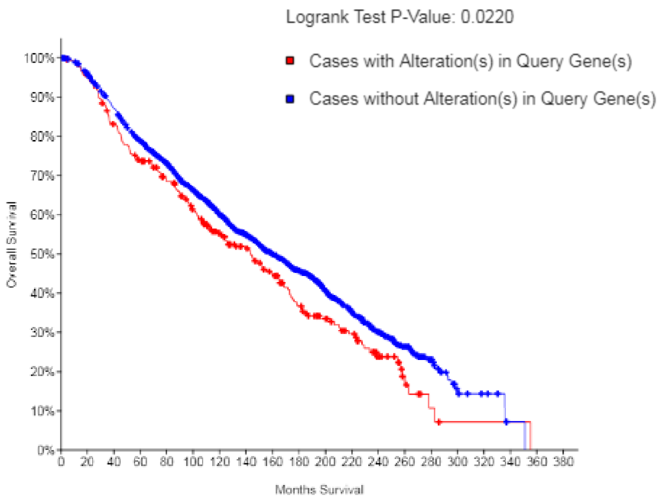

c. Kaplan-Meier survival analysis of patients enrolled in the METABRIC study comparing patients that have gene expression alterations in *SMAD4* and *SNAI2* to those that do not, shown in panel a, and followed for ~ 35 years.

	Number of Cases, Total	Number of Cases, Deceased	Median Month Survival
Cases with Alteration(s) in Query Gene(s)	442	276	146.93
Cases without Alteration(s) in Query Genes(s)	1462	827	159

d. Kaplan-Meier survival analysis of patients enrolled in the METABRIC study having gene alterations (mutations, deletions, amplification) in *SMAD4* and *SNAI2*, compared to survival of all other patients shown in b.

	Number of Cases, Total	Number of Cases, Deceased	Median Month Survival
Cases with Alteration(s) in Query Gene(s)	268	172	143.13
Cases without Alteration(s) in Query Genes(s)	1713	972	159.7

Supplementary Figure legends

Supplementary Figure 1: The effects of microtubule targeting agents on TGF-β-mediated Snail induction. **a**, **b.** mRNA expression analysis of *SNAI1* in MDA-MB-231 and Hs578T cells that were serum-starved for 18 h and then stimulated with TGF-β for the indicated times. Data the average of two independent experiments (± SEM). **c, d.** Western blot analysis of Snail and GAPDH from MDA-MB-231 and Hs578T cells that were serum-starved for 18 h and pretreated with microtubule targeting agents for 2 h followed by a 3 h TGF-β stimulation. **e, f.** mRNA analysis of *SNAI1* transcript in cells that were serum-starved for 18 h and pretreated with microtubule targeting agents for 2 h and then stimulated with TGF-β for either 2 h (MDA-MB-231 cells) or 1 h (Hs578T cells). Data are the average of 3 independent experiments (± SEM). Statistical significance was assessed using a one-way ANOVA with Dunnett's post-hoc test compared to TGF-β-stimulated vehicle controls. (**** $p<0.0001$, *$p<0.05$)

Supplementary Figure 2: Effects of microtubule targeting agents on downstream targets of the TGF-β pathway. **a, b.** Western blot analysis of Smad2/3, Smad4, Snail, Slug, and GAPDH in whole-cell lysates from BT-549 and HCC1937 cells that were treated with microtubule targeting agents for 5 h.

Supplementary Figure 3: Time-dependent nuclear accumulation of Smad2/3 after TGF-β stimulation. **a**, **c.** Immunofluorescence images of Smad2/3 in BT-549 and HCC1937 cells. Cells were serum-starved for 18 h and then stimulated with TGF-β for the times indicated. Images were obtained using the Operetta™ high content imager and are representative of two independent experiments. **b, d.** The percentage of total Smad2/3 present in the nucleus of BT-549 and HCC1937 cells at each time point was quantified using high content imaging. Data are reported for two individual experiments, each comprising 2-3 replicates and reported as mean ± SEM (N=7). Statistical significance compared to control (0 min) was assessed using a one-way ANOVA with Dunnett's post-hoc test (**** $p<0.0001$, *** $p<0.001$, ** $p<0.01$, *$p<0.05$).

Supplementary Figure 4: Effect of microtubule targeting agents on TGF-β-induced nuclear accumulation of Smad2/3. **a**. β-tubulin and Smad2/3 were visualized by immunofluorescence in BT-549 cells. Cells were serum-starved for 18h, pretreated with microtubule targeting agents for 2 h and stimulated with of TGF-β for 45 min. Images were obtained using the Operetta™ high content imager and are representative of four independent experiments. Inset images show magnified view of Smad2/3 localization. **b**. β-tubulin and Smad2/3 localization was evaluated by immunofluorescence in HCC1937 cells. Cells were serum-starved for 18 h, pretreated with microtubule targeting agents for 2 h and stimulated with of TGF-β for 1 h. Images were obtained using the Operetta™ high content imager and are representative of four independent experiments. Inset images show magnified view of Smad2/3 localization.

Supplementary Figure 5: Requirement of Smad4 for TGF-β-mediated Snail induction. **a, c.** Smad4 was transiently knocked-down by siRNAs in MDA-MB-231 and Hs578T cells. Western blot analysis of whole-cell lysates of cells after 18 h of serum-starvation, 2 h pretreatment with microtubule targeting agents followed by 3 h of TGF-β stimulation. Immunoblots were probed for Smad4, Snail, and GAPDH. **b, d.** qRT-PCR analysis of *SNAI1* transcript after siRNA-mediated knock-down of Smad4. Cells were serum-starved for 18 h, pretreated with microtubule targeting agents for 2 h and stimulated with TGF-β for either 2 h (MDA-MB-231) or 1 h (Hs578T). Statistical significance of Smad4 knock-down for each condition was determined using a two-way ANOVA with Holm-Sidak's multiple comparison test. (**** $p<0.0001$, *** $p<0.001$, ** $p<0.01$, *$p<0.05$).

Supplementary Figure 6: Chromatin Immunoprecipitation of *ID1* promoter by Smad4. **a.** Chromatin immunoprecipitation (ChIP) of the *ID1* promoter region bound by Smad4, after 18 h of serum-starvation, pretreatment with either vehicle or eribulin for 2 h followed by stimulation with TGF-β for 1 h in BT-549 cells. qRT-PCR data are reported as percentage of input chromatin for *ID1* promoter sequence and are the mean ± SEM (N=2). Chromatin pulldown with control IgG is also shown. **b.** Smad4 ChIP of *SNAI1* P3 and *ID1* sequences from MDA-MB-468 and BT-549 cells. All cells were serum-starved for 18 h and then either left untreated or stimulated with TGF-β (2 h in MDA-MB-

468 cells and 1 h in BT-549 cells). qRT-PCR data are reported as percentage of input chromatin for the promoter sequences from a single experiment.

Supplementary Figure 7: Effect of microtubule targeting agents on *SNAI2* induction in HCC1937 cells. **a**. qRT-PCR analysis of *SNAI2* mRNA transcript abundance in HCC1937 cells that were serum-starved for 18 h, 2 h pretreated for 2 h with microtubule targeting agents, then stimulated for 2 h with TGF-β. Data are the average of three independent experiments (± SEM) **b.** Western blot analysis of whole-cell lysates from HCC1937 cells that were either serum-starved for 18 h or left in replete conditions and treated with microtubule targeting agents for 5 h. Immunoblots were probed for Slug and GAPDH. **c, d.** qRT-PCR analysis of *SNAI2* transcript in HCC1937 cells that were either serum-starved for 18 h or left in replete conditions and treated with microtubule targeting agents for 4 h. Data represent a single experiment.

Supplementary Figure 8: Impact of Smad2/3/4 knock-down on *SNAI2* expression. **a, b.** Smad4 was transiently knocked-down using siRNAs in BT-549 and HCC1937 cells. qRT-PCR analysis of *SNAI2* mRNA transcript abundance was quantified in cells that were serum-starved for 18 h, pretreated with microtubule targeting agents for 2 and then stimulated with TGF-β (1 h in BT-549 cells and 2 h in HCC1937 cells). Data are the average of two independent experiments (± SEM) and statistical significance of Smad4 knock-down for each condition was determined using a two-way ANOVA with Holm-Sidak's multiple comparison test. (**** $p<0.0001$, *** $p<0.001$, ** $p<0.01$, *$p<0.05$). **c, d.** Smad2/3 proteins were transiently knocked-down in BT-549 and HCC1937 cells using siRNAs. Westerns blot analysis of whole-cell lysates were evaluated after 18 h of serum-starvation, 2 h pretreatment with microtubule targeting agents followed by a 3 h TGF-β stimulation. Immunoblots were probed for Smad2/3, Slug, and GAPDH. These results are from the experiment shown in Fig. 1e, f.

Supplementary Figure 9: Effect of microtubule targeting agents on the expression of the transcription factor c-Jun. **a.** Western blot analysis of BT-549 whole-cell lysates. Cells were serum-starved for 18 h, pretreated with microtubule targeting agents for 2 h, followed by a 3 h stimulation with TGF-β. Immunoblots were probed for total and phosphorylated forms of c-Jun and GAPDH. **b.** Western blot analysis of Smad4 and GAPDH from BT-549 and HCC1937 whole-cell lysates. Immunoblots were probed for Smad4. **c**. Western blot analysis of whole-cell lysates from cells treated with either vehicle or eribulin for 7 days. Immunoblots were probed for Slug, c-Jun, and GAPDH.

Supplementary Figure 10: *SNAI2* expression across breast cancer cell lines. **a**. Breast cancer cell lines were serum-starved for 18 h and treated with vehicle, eribulin or paclitaxel for 4 h. qRT-PCR analysis was performed to evaluate the mRNA expression of *SNAI2*. Data are the average of two independent experiments (± SEM).

Supplementary Figure 11: Evaluation of the METABRIC dataset for alterations in *SMAD4* and *SNAI2*. **a.** Kaplan-Meier survival analysis of patients from the METABRIC study followed up for ~ 35 years comparing patients that have gene expression alternations in *SMAD4* and *SNAI2* to those that do not. **b.** Kaplan-Meier survival analysis of patients in the METABRIC study having genetic alterations (including mutations, deletions, and amplification) in the *SMAD4* and *SNAI2*, compared to survival of all other patients.

Supplementary Table 1

Target	Vendor Information	Application - Dilution
Snail	Cell Signaling Technologies, 3879 (C15D3)	WB – 1:1000
Slug	Cell Signaling Technologies, 9585 (C19G7)	WB – 1:1000
GAPDH	Cell Signaling Technologies, 5174 (D16H11)	WB – 1:1000
Smad2/3	Cell Signaling Technologies, 8685 (D7G7)	WB – 1:1000<br>IF – 1:400
Phospho-Smad2 (Ser465/467)	Cell Signaling Technologies, 3108 (138D4)	WB – 1:1000
Phospho-Smad3 (Ser423/425)	Millipore Sigma, 07-1389	WB – 1:1000
β-Tubulin	Sigma-Aldrich, T4026 (TUB 2.1)	IF – 1:400
Smad4	Abcam, ab40759 (EP618Y)	WB – 1:1000
Smad4	Cell Signaling Technologies, 46535 (D3R4N)	ChIP – 10ul per IP
c-Jun	Cell signaling Technologies, 9165 (60A8)	WB – 1:1000
Phospho-c-Jun	Cell Signaling technologies, 3270 (D47G9)	WB – 1:1000
IgG	Millipore Sigma, 12-370	ChIP – 5ul per IP

WB – Western Blotting; IF – Immunofluorescence; ChIP – Chromosomal Immunoprecipitation

Supplementary Table 2

Target	Primer Sequence
SNAI1	Forward : TCGGAAGCCTAACTACAGCGA<br><br>Reverse : AGATGAGCATTGGCAGCGAG
SNAI2	Forward : CGAACTGGACACACATACAGTG<br><br>Reverse : CTGAGGATCTCTGGTTGTGGT
GAPDH	Forward : ACAACTTTGGTATCGTGGAAGG<br><br>Reverse : GCCATCACGCCACAGTTTC
SNA1 TSS[1]	Forward : GGAGTACTTAAGGGAGTTGGCGG<br><br>Reverse: GAACCACTCGCTAGGCCGT
SNAI1 P3[2]	Forward : TACTTAAGGGAGTTGGCGGC<br><br>Reverse : CGCAGAAGAACCACTCGCTA
ID11	Forward : AGTCCGTCCGGGTTTTATGAAT<br><br>Reverse : CGGTCTGTGTCAGCGTCTGAAC
JUN	Forward : TCCAAGTGCCGAAAAAGGAAG<br><br>Reverse : CGAGTTCTGAGCTTTCAAGGT

Smith, A. P. *et al.* A positive role for Myc in TGFbeta-induced Snail transcription and epithelial-to-mesenchymal transition. Oncogene 28, 422-430, doi:10.1038/onc.2008.395 (2009).
Li, D. *et al.* Role of RbBP5 and H3K4me3 in the vicinity of Snail transcription start site during epithelial-mesenchymal transition in prostate cancer cell. Oncotarget 7, 65553-65567, doi:10.18632/oncotarget.11549 (2016)
